# Editorial: Areawide pest management and agroecosystem resilience to suppress invasive insects

**DOI:** 10.3389/finsc.2025.1605737

**Published:** 2025-04-24

**Authors:** Michael J. Brewer, Seth J. Dorman

**Affiliations:** ^1^ Texas A&M, AgriLife Research, Corpus Christi, TX, United States; ^2^ Forage Seed and Cereal Research Unit, USDA ARS, Corvallis, OR, United States

**Keywords:** agroecology, sustainable pest management, natural enemies, pest suppression, resilient agroecosystems, cultural control, editorial

This Research Topic contributes to the field of areawide pest management, with a focus on assessment of pest risk and agroecosystem resilience to pests in the context of spatial variability of pest activity and natural pest suppression across an agroecosystem. The areawide pest management concept as initially put forward emphasizes the use of pest management practices across a region with the intent to reduce or eliminate target pests areawide (1). Areawide pest suppression is unlikely to be achieved when integrated pest management (IPM) practices are implemented at the field scale based on insect monitoring and use of economic thresholds in individual fields (2, 3). Areawide suppression of pest populations reduces the likelihood and severity of reintroduction from source hosts (crop and non-crop). This concept is particularly applicable to invasive species that enter into an agroecosystem with attributes beneficial to pest biology and depauperate of suppression agents, resulting in substantial agricultural disruption over a large area (1). Further, an areawide approach may support IPM strategies by reducing the abundance of key pest populations at broader geographic scales thereby enabling the management of secondary pests with non-chemical approaches. This Research Topic extends this concept to address spatial variability of invasive as well as perennial pests and their suppression agents such as natural enemies. Traditionally, spatial and temporal variations of pest populations, including invasive species, have been documented along temperature gradients and among different crops and cultivars of a crop in the IPM literature (2), and through the action of natural enemies (4). More recently in the expanding agroecology literature, the influence of additional landscape elements on pests and natural enemies has been considered (5, 6).

An agroecosystem orientation for management of invasive species becomes more valuable when source-sink insect dynamics, insect movement, habitat affiliation, pest suppression agents, and crop sensitivity to the pest vary spatially across a crop production region (3). This variability is consistent with the viewpoint that pest and natural enemy abundance and species diversity are spatially variable and conditional on the range of agricultural (inclusive of crop genetics, crop field size and shape, and crop diversity) and environmental (inclusive of weather and climate, and local to regional semi-natural vegetative structure) conditions that themselves vary in the landscape (3, 7–9). Overall, the goal of this Research Topic is to document examples of areawide pest management approaches to assess pest risk along with pest suppression that is naturally occurring in an agroecosystem and target insertion of areawide pest management practices that improve pest suppression.

As an example, the landscape and weather of an agroecosystem are spatially and temporarily heterogeneous in the North American Great Plains even though a few cereal crops dominate the cropping system. This off-crop heterogeneity is associated with varying capacity to suppress several cereal aphid invaders (10) and most recently the sorghum aphid, *Melanaphis sorghi* (Hemiptera: Aphididae) (Brewer et al.). Using spatially explicit ecological modeling in this system, Koralewski et al. found the extent and form of heterogeneity are relevant to natural pest suppression, including landscape elements, weather conditions, and the temporal sequence of arrival of mobile aphids and activity of their natural enemies. In considering weather and insect movement across a large grass seed and vegetable agroecosystem, Slone et al. focused on *Agrotis ipsilon* (Lepidoptera: Noctuidae). The spatiotemporal occurrence of *A. ipsilon* was forecast using phenological models taking into account temperature-dependent population growth which varies by crop host and the moth’s migratory behavior across the system. Brewer working in the cotton agroecosystem further demonstrated that distance metrics (nearest distance of non-crop habitat of pests to cotton fields) were useful in evaluating early season risk of a plant bug, *Creontiades signatus* (Hemiptera: Miridae), infesting cotton. The value of distance was previously used to recommend planting distances of cotton to alfalfa as a means to reduce the threat of another mirid species, *Lygus hesperus*, infesting cotton from source alfalfa fields that when cut induced movement of *L. herperus* to nearby cotton fields (11). In these examples, the associations with spatially variable pest abundance were revealed by applying landscape ecology principles and advances in geographic information systems (GIS) to archive, manipulate, and analyze spatial insect, landscape, and weather data, with key associations verified by experimental manipulation when possible.

Regarding the application of management practices areawide, Herreid et al. used a simple agronomic harvest practice in alfalfa hay production (i.e., cutting the first hay crop early) to suppress alfalfa weevil, *Hypera postica* (Coleoptera: Curculionidae). Cutting alfalfa early decreased weevil populations directly with no apparent disruption to parasitism rates of alfalfa weevil through the harvest event. *Hypera postica* and its Ichneumonid parasitoid are common across the Intermountain western United States, providing the rationale for applying early first cut of hay areawide. Additional examples can be found in the areawide pest management literature (e.g., 12) and literature focusing on specific pest suppression techniques applied areawide (e.g., 13).

We hope the articles posted to this Research Topic stimulate further research into using spatial information of landscape, weather, and pest suppression agents to assess pest risk region-wide and target insertion of areawide pest management practices that improve pest suppression where it is lacking in the near-term and may bolster agroecosystem resilience to pests in the long-term.
